# The impact of BRCA mutation and hormone receptor status on the outcomes of fertility preservation in breast cancer patients: a systematic review and meta-analysis

**DOI:** 10.3389/fonc.2025.1639420

**Published:** 2025-11-28

**Authors:** Liyu Ye, Weihui Yang, Huiyuan Guan

**Affiliations:** Department of Breast Surgery, Huzhou Maternity & Child Health Care Hospital, Huzhou, Zhejiang, China

**Keywords:** fertility preservation, breast cancer, brca mutation, hormone receptor status, ovarian stimulation

## Abstract

**Background:**

Fertility preservation is a critical aspect of care for young breast cancer (BC) patients undergoing gonadotoxic treatments. BRCA mutation and hormone receptor (HR) status influence tumor biology and treatment outcomes. This study evaluated the impact of BRCA mutation and HR status on fertility preservation outcomes in BC patients.

**Methods:**

PubMed, Embase, Scopus, and Web of Science databases were searched for publications from inception to March 31, 2025 that report on fertility preservation outcomes stratified by BRCA mutation or HR status. Primary outcomes included the number of retrieved oocytes, maturation rates, and ovarian reserve indices such as anti-Müllerian hormone (AMH) levels and antral follicular count (AFC). Random-effects meta-analyses were performed.

**Results:**

Thirteen studies involving approximately 1,654 participants were included in the meta-analysis. Patients with no BRCA mutations reported significantly higher mature oocytes (MD: -1.48, 95% CI: -2.63 to -0.34) compared to those with BRCA mutations and non-significant total oocyte yield (MD: -1.37, 95% CI: -3.13 to 0.40). AFC and AMH levels showed no significant intergroup differences. Additionally, estrogen receptor (ER)-positive patients exhibited better ovarian response, with higher AFC (MD: 1.37, 95% CI: 0.48 to 2.26) and greater oocyte yield (MD: 1.35, 95% CI: 0.67 to 2.02).

**Conclusion:**

Our results show that BRCA mutations may be associated with significantly diminished mature oocyte production during fertility preservation in BC patients. On the contrary, ER-positive status seems to be associated with high AFC and oocyte yield indicating a more advantageous ovarian response. The present findings are from a limited number of heterogenous studies and hence must be interpreted with caution.

**Systematic Review Registration:**

https://www.crd.york.ac.uk/prospero/, identifier CRD42025641361.

## Introduction

Breast cancer (BC) is the most prevalent form of malignancy in women of reproductive age (1). The hormonal receptor (HR) profile and mutation state of BC, in addition to tumor grade and stage, may provide critical information about the aggressiveness and evolution of the disease (2, 3). Estrogen receptors (ER), progesterone receptors (PR), or human epidermal growth factor receptor 2 (HER2) expression has significant implications for prognosis and the selection of therapeutic modalities and is routinely used for tumor classification (4, 5). ER-positive (ER+) tumors are responsive to anti-hormonal therapies like selective estrogen receptor modulators or aromatase inhibitors, while ER-negative (ER−) tumors are usually more aggressive and respond less to hormonal therapies (6, 7). Triple-negative breast cancer, lacking ER, PR, and HER2 expression, carries a poorer prognosis compared to ER+ or HER2-enriched tumors (8).

In many countries, the evaluation of ER, PR, and HER2 status is routinely incorporated into diagnostic workflows, serving not only as key biomarkers for guiding therapy but also as important prognostic and monitoring tools. Specific mutations and expression patterns of these receptors have been shown to correlate with mammographic findings, thereby enhancing diagnostic accuracy and disease surveillance. On mammograms, ER/PR-positive tumors typically appear as spiculated, low-density masses, whereas HER2-positive and triple-negative malignancies are more commonly associated with pleomorphic calcifications, irregular high-density masses, or without distinguishing features despite aggressive behavior. Integrating imaging characteristics and receptor profiling has been shown to improve diagnosis accuracy and prognosis in BC care (9, 10).

Other than these receptors, genetic mutations, such as those in the BRCA1 and BRCA2 genes, also play a pivotal role in BC pathogenesis (11). BRCA genes belong to the family of ATM-mediated DNA double-strand break repair genes, essential for maintaining genomic stability and telomere integrity. BRCA1 and BRCA2 mutations in females are linked to a significantly increased risk of developing breast and ovarian cancers, often at a younger age and before menopause (12). Moreover, BRCA mutations not only influence BC prognosis but may also affect reproductive outcomes and ovarian reserve, further complicating treatment planning in young patients (13).

With current advances in oncology, the long-term survival rates of young women with BC have considerably improved, reaching as high as 85–90% (14). As survival rates improve, the ability to bear children post-treatment has become a critical consideration in therapeutic planning, shifting the focus towards fertility preservation (15). Consequently, BC patients represent the majority of individuals seeking oocyte and embryo cryopreservation today (16). Fertility preservation strategies, including cryopreservation of oocytes or embryos, are essential for mitigating the gonadotoxic effects of chemotherapy and radiation (17).

However, the impact of the HR status and BRCA mutation status on the fertility preservation outcomes remains unclear. ER and PR status were shown to directly impact tumor biology and treatment, which may, in turn, affect the ovarian response to stimulation during fertility preservation procedures (18). Similarly, BRCA mutations, which may alter ovarian reserve and function, could influence the number and quality of retrieved oocytes (19). According to the NCCN (National Comprehensive Cancer Network) guidelines, fertility preservation should be discussed with all reproductive-aged women at diagnosis, ideally before initiation of systemic therapy (20). The recommendations emphasize early referral to reproductive specialists and the use of ovarian stimulation protocols adapted to HR status, such as letrozole-based regimens for ER-positive patients, to balance oncologic safety with fertility outcomes. However, there remains a deficiency in literature quantifying the impact of BRCA mutation and HR status on fertility outcomes. There have been a prior review examining fertility outcomes in BRCA carriers (12) but with limited data on BC patients. A recent updated review has also summarized evidence on the impact of BRCA mutations on fertility outcomes but without a quantitative analysis (18). Given this deficiency in literature, we conducted this present systematic review and meta-analysis to evaluate whether HR status (ER+, PR+) and BRCA mutation status affect fertility preservation outcomes in BC patients.

## Materials and methods

This study adhered to the Preferred Reporting Items for Systematic Reviews and Meta-Analyses (PRISMA) guidelines (21). The protocol for performing this review was framed *a priori* and was registered in PROSPERO (CRD42025641361).

### Research question

This review addressed the research question: Do HR status and BRCA mutation status influence fertility preservation outcomes in BC patients?

To frame this question, the PICO model was applied:

Population (P): Women diagnosed with BC who underwent fertility preservation procedures.

Exposure (E): BRCA+ or HR+ (ER or PR) status.

Comparison (C): BRCA- or HR- (ER or PR) status.

Outcome (O): Fertility metrics including the number of retrieved oocytes, oocyte maturation rates, anti-Müllerian hormone (AMH) levels and antral follicular count (AFC).

### Search strategy

Digital searches were conducted across PubMed, Embase, Web of Science, and Scopus databases for studies published up to March 31, 2025. The following search string was developed and applied, using keywords and Medical Subject Headings (MeSH): (“fertility preservation” OR “oocyte cryopreservation” OR “embryo cryopreservation”) AND (“BRCA mutation” OR “BRCA1” OR “BRCA2”) AND (“hormone receptor status” OR “ER positive” OR “PR positive” OR “triple-negative breast cancer” OR “TNBC”) AND (“breast cancer”). The search strategies for individual databases are provided in **Table 1**. Additionally, reference lists of eligible studies were reviewed manually to ensure no relevant articles were overlooked.

**Table 1:** Search Strategies for Digital Databases.

Database	Search string	# Records
Pubmed	(“fertility preservation” OR “oocyte cryopreservation” OR “embryo cryopreservation”) AND (“BRCA mutation” OR “BRCA1” OR “BRCA2”)	92
Embase	(‘fertility preservation’/exp OR ‘fertility preservation’ OR ‘oocyte cryopreservation’/exp OR ‘oocyte cryopreservation’ OR ‘embryo cryopreservation’/exp OR ‘embryo cryopreservation’) AND (‘BRCA mutation’/exp OR ‘BRCA mutation’ OR ‘BRCA1’/exp OR ‘BRCA1’ OR ‘BRCA2’/exp OR ‘BRCA2’) AND (‘hormone receptor status’/exp OR ‘hormone receptor status’ OR ‘estrogen receptor positive’/exp OR ‘ER positive’ OR ‘progesterone receptor positive’/exp OR ‘PR positive’ OR ‘triple-negative breast cancer’/exp OR ‘TNBC’ OR ‘HER2 positive’/exp OR ‘HER2 negative’) AND (‘breast cancer’/exp OR ‘breast neoplasm’ OR ‘breast carcinoma’ OR ‘breast tumor’ OR ‘mammary carcinoma’)	21
Web of science	TS= (“fertility preservation” OR “oocyte cryopreservation” OR “embryo cryopreservation”) AND TS= (“BRCA mutation” OR “BRCA1” OR “BRCA2”)	117
Scopus	(“fertility preservation” OR “oocyte cryopreservation” OR “embryo cryopreservation”) AND (“BRCA mutation” OR “BRCA1” OR “BRCA2”) AND (“hormone receptor status” OR “ER positive” OR “PR positive” OR “triple-negative breast cancer” OR “TNBC”) AND (“breast cancer”).	226

The search results were imported into a citation management tool to organize references systematically. Citation manager’s automated tools were used for deduplication, followed by manual verification to ensure accuracy.

### Study selection

In the first stage, the two independent authors screened the titles and abstracts of all identified studies for eligibility. Studies meeting the requirements were advanced to the second stage of full-text assessment for eligibility.

### Inclusion criteria

- Studies involving women diagnosed with BC who underwent fertility preservation procedures such as oocyte or embryo cryopreservation were included.
- Studies reporting outcomes of fertility preservation techniques, including ovarian stimulation, oocyte retrieval, and embryo cryopreservation.
- Studies reporting at least one of the following fertility-related outcomes: Number of oocytes retrieved, Oocyte maturation rates, AMH levels, AFC, etc.
- Prospective or retrospective cohort studies, case-control studies, or randomized controlled trials (RCTs).
- Articles published in English.

### Exclusion criteria

- Studies lacking data on fertility-related outcomes (e.g., oocyte yield, maturation rates, fertilization rates).
- Case reports, case series with fewer than 10 participants, or review articles.
- Studies with duplicate data published in multiple articles.

All disagreements were resolved by discussion between authors or with a third reviewer. All included studies were required to report on fertility preservation outcomes stratified by HR status or BRCA mutation status in BC patients.

### Data extraction

A standardized data extraction form was generated to collect study characteristics (e.g., authors, publication year, study design, sample size), patient data (e.g., age, BRCA mutation status, HR status), and fertility preservation outcomes (e.g., number of retrieved oocytes, oocyte maturation rates, rates of fertilization and pregnancy).

Two independent reviewers extracted data and resolved all discrepancies by discussion. A third reviewer verified the data extraction to ensure accuracy.

### Data synthesis and quality assessment

The quality of the studies was assessed by the Newcastle-Ottawa Scale (NOS) that is scoring the selection of study groups, comparability, and ascertainment of outcomes, with a maximum score of 9, and higher scores indicating better study quality (22).

Quantitative data were synthesized using meta-analytical techniques when possible. The meta-analysis used RevMan (version 5.4, The Cochrane Collaboration, UK). Pooled analyses were conducted using random-effects models to account for heterogeneity between studies. The continuous data was expressed as mean and standard deviation (SD). Data expressed as median and range were converted into mean and SD, after considering the sample size according to the provided equation (23). Statistical heterogeneity was evaluated using the I² statistic. I^2^ of 25%, 50%, and 75% indicated low, moderate, and high heterogeneity, respectively. Where meta-analysis was not feasible, a narrative synthesis of findings was performed.

### Publication bias

Visual inspection of Funnel plots was carried out to assess publication bias.

## Results

### Search results

**Figure 1:**
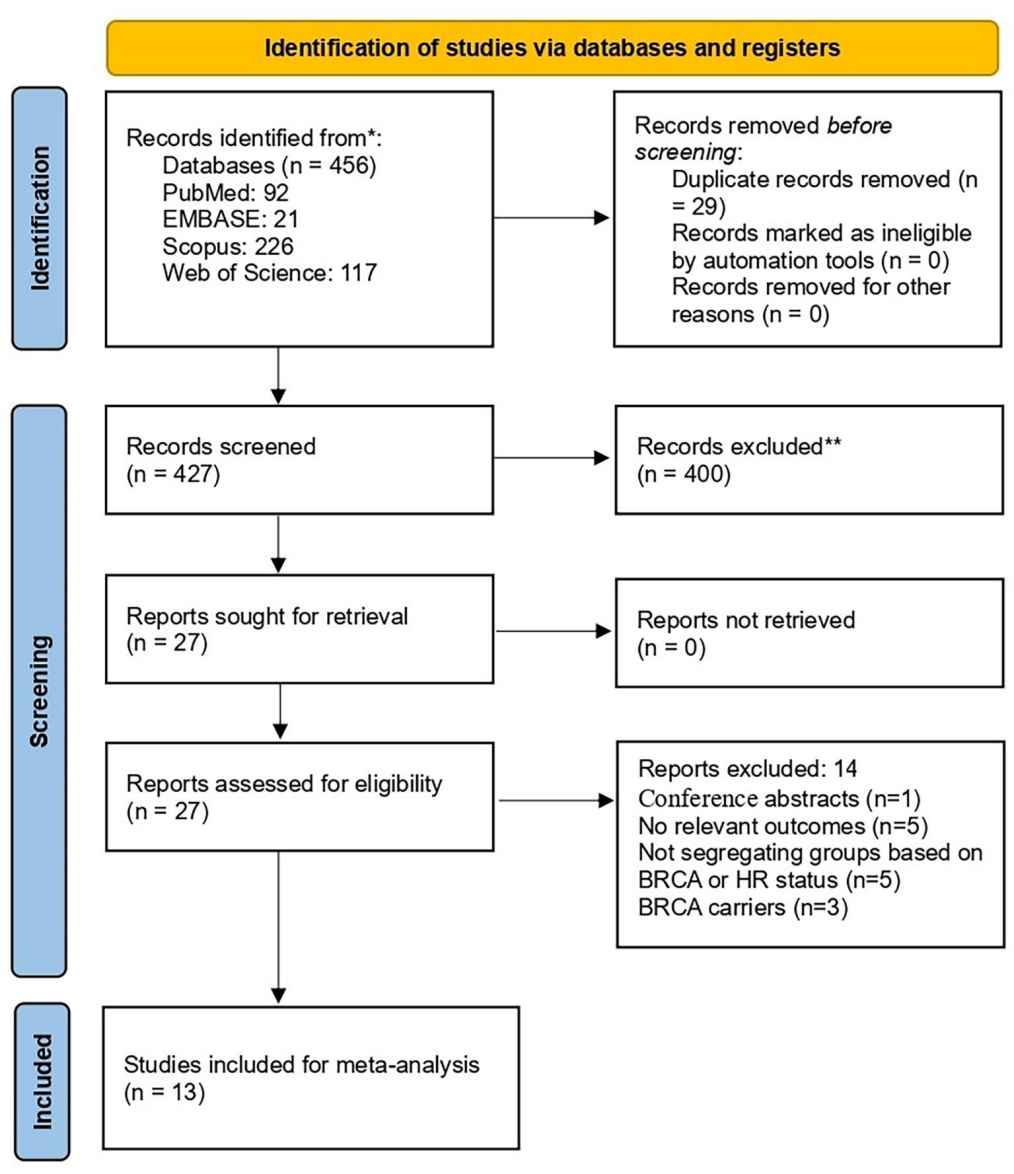
Study selection flow chart.

The initial search retrieved 456 records. After removing the duplicates, 427 underwent title and abstract screening. Subsequently, full-text evaluation of eligibility was carried out for 27 articles. Finally, thirteen articles (24–36) were included (**Figure 1**).

### Baseline details

A total of thirteen studies published between 2010 and 2024 were included, comprising both retrospective (n=10) and prospective (n=3) cohort designs, with sample sizes ranging from 29 to 329 participants. The total sample size of all studies was 1,654. The majority of studies were conducted in Europe and North America, with few studies from Asia and Australia. The mean age of participants was approximately 31–35 years across studies. Majority studies evaluated women with BC carrying BRCA1/2 mutations and only three studies stratified patients based on ER status. Fertility preservation was consistently performed prior to initiation of systemic therapy in all studies. The ovarian stimulation protocols varied among studies. Random-start gonadotropin-releasing hormone (GnRH) was the most frequently used stimulation protocol (24, 36, 37). Letrozole combined with GnRH antagonists was also commonly employed (34, 35, 38). Recombinant follicle-stimulating hormone (FSH) with or without human menopausal gonadotropin (hMG), was widely used (26, 28, 31). The outcomes reported by the studies also showed wide variation. The NOS quality scores ranged from 7 to 8, indicating generally moderate-to-high methodological quality. **Table 2** provides the characteristics and quality of the individual studies.

**Table 2:** General characteristics of included studies.

Author and Year	Country	Study design	Sample size	Age (years)	Hormone Receptor/BRCA mutation status (n)	BRCA mutation type	Ovarian stimulation	Serum AMH levels, ng/mL	Timing of fertility preservation	Outcome	Adjusted for	NOS score
Liu et al., 2024 (38)	China	R	47	31.5 ± 4.4	ER + (36) ER – (11)	NR	Letrozole, Random start Gonadotropin-releasing hormone antagonist (or) progestin-primed ovarian stimulation protocols	NR	Before treatment	Number of mature oocytes; numbers of total oocytes retrieved, peak estradiol levels, and subsequent fertility preservation	NR	7
El Moujahed et al., 2023 (24)	France	R	311	33.4 (30.5–36)	BRCA (57) Non-BRCA (254)	BRCA1 and BRCA2	Random start gonadotrophin releasing hormone	1.6 (0.8–2.9) ng/mL	Before treatment	No of follicles ≥16 mm, No of oocytes retrieved, No of metaphase II oocytes, Oocyte Retrieval Rate (%), Oocyte maturation rate (%), FORT* (%)	NR	8
Prokurotaite et al., 2023 (25)	Belgium	R	75	32.2 ± 3.9	gBRCAPV (20) gBRCAPV (55)	gBRCAPV	Standard, Random follicular, Random luteal	1.9[0.2–13] µg/L	Before treatment	Number of oocytes collected, Maturation rate, Total number of cryopreserved oocytes, Total number of oocytes fertilized, Fertilization rate	NR	8
Sii et al., 2023 (26)	Australia	R	214	ER**+ =** 35.0 (34.3– 35.7); ER - **=** 33.4 (32.1– 34.8)	ER + (154) ER – (60)	NR	Daily recombinant follicle- stimulating hormone	NR	Before treatment	Total number of oocytes frozen; total number of oocytes collected, mature oocytes, and embryos frozen	NR	7
Kim et al., 2022 (27)	South Korea	R	59	33.3	BRCA (39) Non-BRCA (20)	BRCA1 and BRCA2	Recombinant human chorionic gonadotropin	4.2 ± 3.6 ng/mL	Before treatment	Retrieved oocytes and mature oocytes	NR	7
Balayla et al., 2020 (28)	Canada	R	155	32 (28–35)	ER/PR + (97) ER/PR – (58)	BRCA	Recombinant follicle-stimulating hormone, Human menopausal gonadotropin	NR	Before treatment	Total number of mature oocytes; number of retrieved oocytes, serum estradiol levels, and number of follicles > 14 mm	Age	7
Ponce et al., 2020 (29)	Spain	R	135	BRCA -ve = 41.6 ± 7.6, BRCA1 = 41.4 ± 6.4, BRCA2 = 41.1 ± 6.4	BRCA (69) Non-BRCA (66)	BRCA1 and BRCA2	NR	BRCA -ve = 2.27 ± 2.03 ng/ml, BRCA1 = 3 ± 2.27 ng/ml, BRCA2 = 2.54 ± 2.07 ng/mL	Before treatment	Childbirths, Spontaneous abortion, Nulliparous	Age, BMI, duration of birth control, smoking, gravity, parity, and age >35	8
Porcu et al., 2020 (30)	Italy	P	46	BRCA1+: 31.5 ± 3.2, BRCA2+:33.2 ± 4.5 and BRCA-32.5 ± 4.3	BRCA (22) Non-BRCA (24)	BRCA1+, BRCA2+ and BRCA-	Gonadotropin	NR	Before treatment	Developed follicles, Retrieved oocytes, Cryopreserved oocytes MII	Age, BMI	8
Gunnala et al., 2019 (31)	USA	R	91	BRCA = 32.4 ± 3.6; Non-BRCA = 32.5 ± 4.3	BRCA (38) Non-BRCA (53)	BRCA1 and BRCA3	Follicle-stimulating hormone, Human menopausal gonadotropin	NR	Before treatment	Antral follicles count, Anti-mullerian hormone, day-3 follicle stimulating hormone level, number of harvested oocytes, and number of mature/cryopreserved oocyte	Age, BMI	8
Grynberg et al., 2019 (32)	France	R	329	32.1 ± 3.8	BRCA (52) Non-BRCA (277)	BRCA+ (BRCA1, BRCA2) and BRCA-	NR	4.0 ± 3.5ng/mL	Before treatment	Number of COC retrieved, Oocyte Retrieval Rate, Maturation rate after 48, Number of M-II oocyte	NR	7
Lambertini et al., 2018 (33)	Belgium	R	29	31 (28 – 33)	BRCA (10) Non-BRCA (19)	BRCA1, BRCA2	Follicular and random	1.8 (1.0 -2.7) µg/L	Before treatment	Number of oocytes, Number of mature oocytes, Maturation rate, Number of cryopreserved oocytes, Poor response rate	NR	7
Turan et al., 2018 (34)	USA	P	118	NR	BRCA (21) Non-BRCA (97)	BRCA + and BRCA -	Letrozole combined with recombinant follicle-stimulating hormone	NR	Before treatment	Total number of oocytes and embryos obtained per cycle	Age, BMI, baseline follicle stimulating hormone level, and BRCA status	8
Oktay et al., 2010 (35)	USA	P	45	33.1± 2.8	BRCA (12) Non-BRCA (33)	BRCA1, BRCA2	Letrozole and gonadotropins	NR	Before treatment	NR	Age	7

R, retrospective cohort; P, prospective cohort; NR, not reported; AMH, anti-müllerian hormone; gBRCAPV, Germline BRCA1–2 pathogenic variants; BRCA, BReast CAncer gene; NOS, Newcastle Ottawa Scale; n, number of patients.

### BRCA mutation

**Figure 2:**
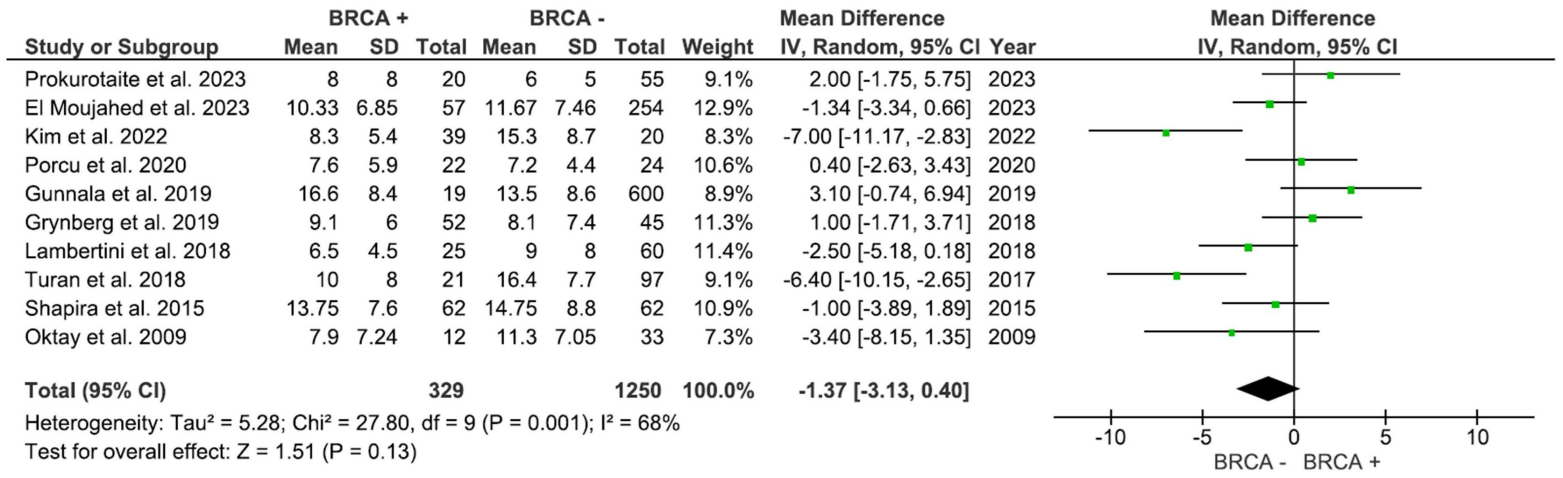
Number of oocytes retrieved based on BRCA mutation status.

A total of 10 studies with 1,579 participants compared the number of retrieved oocytes in women with and without BRCA mutations (**Figure 2**). The pooled analysis showed no statistically significance (p=0.13), with an MD of -1.37 (95% CI: -3.13 to 0.40), in the number of oocytes in the Non-BRCA group (I^2^ = 68%).

**Figure 3:**
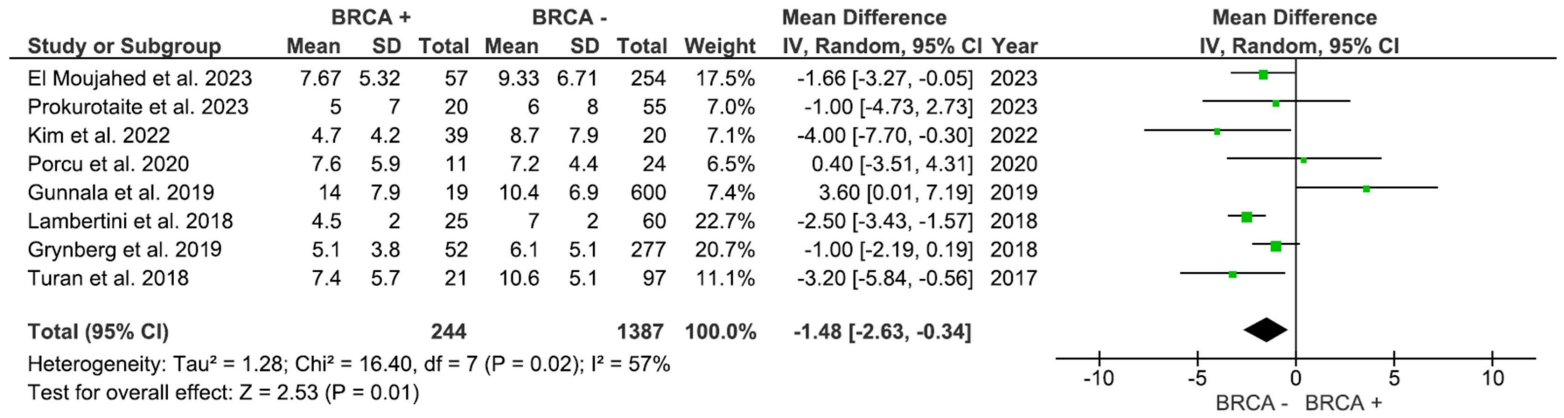
Number of mature oocytes based on BRCA mutation status.

The number of retrieved mature oocytes was reported in 8 studies comprising 1,631 participants. As shown in **Figure 3**, the number of mature oocytes was markedly higher in the non-BRCA group (MD: -1.48, 95% CI: -2.63 to -0.34), p=0.01 (I^2^ = 57%).

**Figure 4:**
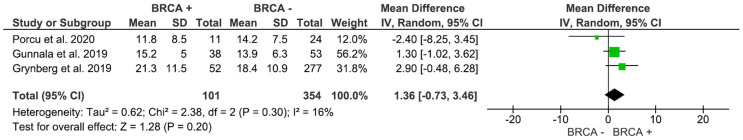
Antral Follicular count based on BRCA mutation status.

**Figure 5:**
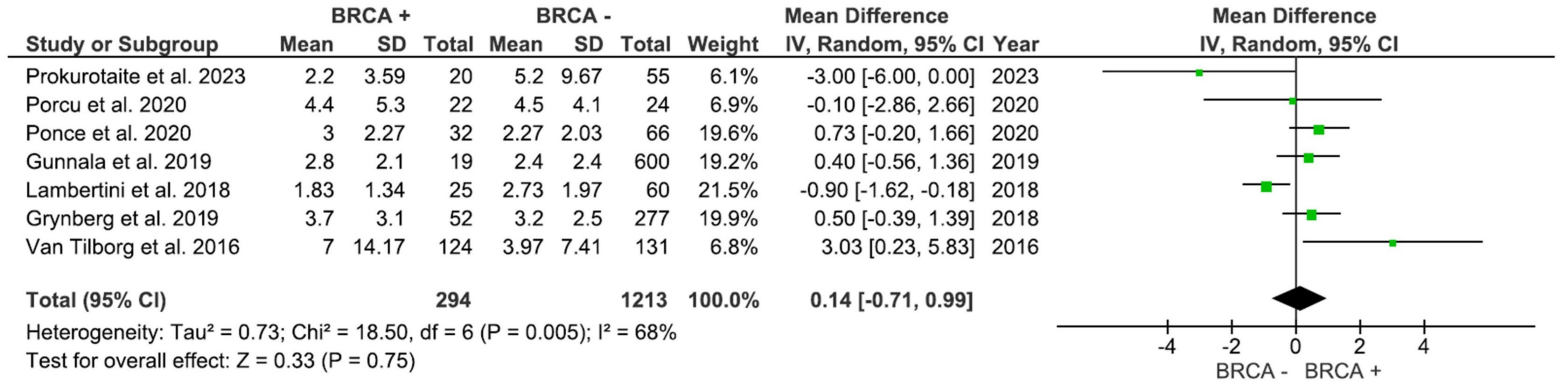
AMH levels based on BRCA mutation status.

AFC, assessed in 3 studies with 455 participants, was higher in individuals with BRCA mutations compared to patients with no mutations (**Figure 4**); however, the result was not significant (MD: 1.36, 95% CI: -0.73 to 3.46), p=0.20 (I^2^ = 16%). As shown in **Figure 5**, AMH levels, pooled from 7 studies including 1,507 participants, was not significantly different between BRCA and non-BRCA BC patients (MD: 0.14, 95% CI: -0.71 to 0.99), p=0.75 (I^2^ = 68%).

### ER status

**Figure 6:**
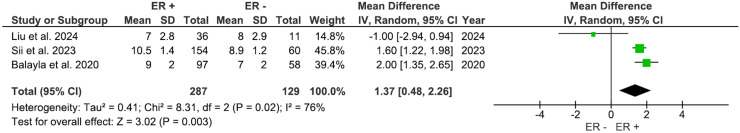
AFC based on estrogen receptor status.

**Figure 7:**
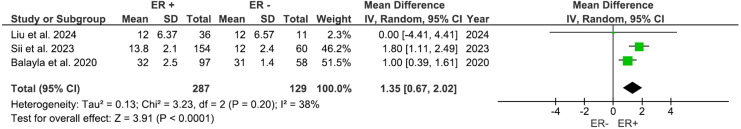
Number of oocytes retrieved based on ER receptor status.

Three studies, including 416 participants, evaluated outcomes based on the ER status. AFC was significantly higher (**Figure 6**) in the ER-positive group compared to other groups, with a mean difference of 1.37 (95% CI: 0.48 to 2.26), p=0.003 (I^2^ = 76%). The number of retrieved oocytes was also considerably higher in the ER-positive group (**Figure 7**), with a mean difference of 1.35 (95% CI: 0.67 to 2.02), p<0.0001 (I^2^ = 38%).

## Discussion

This systematic review and meta-analysis analyzed data from 13 studies involving over 1,654 BC patients undergoing fertility preservation to assess the impact of BRCA mutation status and HR status, specifically ER expression, on ovarian response and fertility outcomes. Our data show that BRCA mutation carriers have considerably fewer mature oocytes and a tendency of lower total oocytes as compared to non-carriers. The meta-analysis also showed that ER-positive patients have a superior ovarian response, as measured by both AFC and oocyte yield.

Our findings augment and expand upon previous reviews on this subject. Hong et al. (17) have previously presented a comprehensive narrative overview of fertility preservation in young women with BC, emphasizing the challenges associated with tumor biology, gonadotoxic therapies, and the necessity for personalized stimulation protocols, yet refraining from quantitatively assessing the influence of BRCA mutations or HR status. Dias Nunes et al. (18) specifically examined BRCA mutations and concluded that BRCA carriers, especially those with BRCA1, may have reduced ovarian reserve and potentially poorer oocyte yield. Nevertheless, their conclusions mostly relied on AMH and AFC data instead of aggregated fertility preservation outcomes. Our results are also consistent with the earlier meta-analysis by Gasparri et al. (12), which examined ovarian reserve markers in women with and without BRCA pathogenic variants. Their study reported lower AMH levels among BRCA1 mutation carriers, suggesting an accelerated decline in ovarian reserve. However, their review could include just two studies specific to BC patients. Our results support these findings by indicating that carriers of BRCA mutations among BC patients have impaired ovarian function. However, we also add to the body of data by demonstrating substantial reductions in retrieved and mature oocytes, which are more clinically significant fertility preservation outcomes. Furthermore, in contrast to previous studies (12, 17, 18), our study distinctly includes ER status, providing a more comprehensive overview of evidence on impact of both BRCA mutation and receptor status on fertility outcomes of BC patients.

The pooled analysis indicated that carriers of the BRCA mutation exhibited a tendency of diminished oocyte yield and significantly lower maturation rates. In contrast, AMH levels and AFC did not exhibit significant differences between the groups. It is crucial to remember that AMH and AFC may understate the qualitative effect of BRCA mutations on oocyte competence and are not perfect indicators of reproductive capacity (39). On the pathophysiological perspective, it seems plausible for BRCA mutations to affect oocyte production and maturation rates due to the pivotal function of BRCA1 and BRCA2 in the homologous recombination repair of DNA double-strand breaks (DSBs). Loss-of-function mutations hinder the identification and repair of double-strand breaks (DSBs) in oocytes, hence expediting follicular atresia and ovarian aging (35, 40). At the cellular level, BRCA-deficient oocytes exhibit impaired RAD51 loading and accumulate unrepaired DSBs, which are indicated by elevated γ-H2AX foci. This results in checkpoint failure and increased primordial follicle apoptosis (41, 42). In addition to repairing DSBs, BRCA proteins preserve telomere integrity and regulate replication forks and therefore their absence can fosters genomic instability, meiotic errors, and aneuploidy during oogenesis (43, 44). Our findings extend these molecular insights, illustrating that DNA repair failure in BRCA carriers significantly influences clinically relevant fertility preservation outcomes, including oocyte yield and maturation.

Our meta-analysis also demonstrated that ER-positive patients exhibit higher AFC and greater oocyte retrieval compared with ER-negative patients. These results may be explained by several mechanisms. By primarily regulating granulosa cell proliferation, differentiation, and gonadotropin sensitivity, estrogen signaling plays a crucial and permissive role in folliculogenesis. Estrogens synthesized by granulosa cells bind with ERs (ERα and ERβ) in granulosa and theca cells, enhancing FSH-induced follicle growth, antral follicle survival, and oocyte maturation (45, 46). Mechanistically, ER activation upregulates genes implicated in granulosa cell proliferation. It also enhances FSH receptor expression and downstream cAMP signaling, thereby increasing the population of follicles responsive to exogenous gonadotropin stimulation during controlled ovarian stimulation (45, 46). As a result, patients with ER-positive tumors probably have a systemic endocrine environment (or preserved intragonadal estrogen signaling) that is better for recruiting and maturing antral follicles. This is in line with the higher AFC and oocyte yields we found in our meta-analysis. In addition to direct impacts on follicles, clinical and logistical considerations associated with ER positive status can enhance this biological advantage. ER-positive BC are frequently identified at earlier stages, facilitating the implementation of planned, letrozole-enhanced stimulation protocols that decrease peak circulating estradiol levels while preserving follicular response. Studies have shown that letrozole co-treatment sustains oocyte yield while minimizing estrogen exposure (47), a methodology widely adopted and endorsed in modern fertility-preservation practices. In contrast, ER-negative and triple-negative cancers require more urgent systemic therapy, which can narrow the window for optimum stimulation and force shorter or changed protocols that diminish oocyte yield (45, 47, 48). These biological (ER-mediated folliculogenesis) and pragmatic (timing and protocol choice) factors likely elucidate the superior ovarian response observed in ER-positive individuals compared to their ER-negative counterparts in our pooled analysis.

While the aim of this review was to assess the impact of all types of HR status and fertility outcomes, our results were limited to ER only due to paucity of data on PR or HER2 expression. BC with HER2 is often seen in young women and is aggressive in nature with poor patient survival (49). Literature suggests that HER2 expression may be associated with reduced oocyte maturation rate but data remains limited (50). Likewise, therapies specifically targeting HER2 like trastuzumab are being widely used but with limited data on their impact on fertility. Animal studies have shown that trastuzumab effectively mitigated vascular damage and apoptosis induced by cyclophosphamide and paclitaxel, leading to an increased ovarian reserve post-treatment and indicating a potential protective effect (51). However, how these therapies affect human fertility outcomes remain to be studied.

The timing of fertility preservation in young BC patients continues to be a persistent issue. The effects of cancer treatment on ovarian reserve and fertility outcomes contrast with concerns over the influence of controlled ovarian stimulation and assisted reproductive technologies on disease outcomes. In all included studies, fertility preservation was conducted prior to the commencement of anti-cancer therapy due to the established toxicity of BC treatment. Chemotherapy is highly deleterious, directly harming oocytes, diminishing antral follicle count and anti-AMH levels, and causing treatment-related amenorrhea or premature ovarian insufficiency (52, 53). Hormonal medications, notably long-term tamoxifen, may not directly impair ovarian reserve, but they do delay childbirth and lead to transitory amenorrhea, limiting the reproductive window (54, 55). Novel targeted agents, including PARP and CDK4/6 inhibitors, pose further issues by compromising follicular integrity and granulosa cell functionality (56, 57), whereas immunotherapies and checkpoint inhibitors may induce primary or secondary hypogonadism (58, 59). Significantly, findings from an extensive meta-analysis demonstrate that fertility preservation methods—such as controlled ovarian stimulation, oocyte and embryo cryopreservation, and assisted reproductive technologies—are oncologically safe, exhibiting no heightened recurrence risk and even a tendency toward enhanced outcomes, including diminished recurrence and mortality rates (60). Additionally, this advantageous trend was noted in HR–positive subgroups and among patients undergoing neoadjuvant chemotherapy. Collectively, these data emphasize the necessity of including fertility preservation into treatment planning at an early stage, while ensuring both patients and physicians of its safety in both pre- and post-treatment contexts.

This study has some limitations. There was a heterogeneity in study design, participant demographics, ovarian stimulation protocols, and fertility outcomes. This heterogeneity limited the ability to draw definitive conclusions and underscores the need for standardization in future research. Despite this limitation, the comprehensive approach and adherence to PRISMA guidelines enhance the reliability of the review. Many studies did not adequately adjust for patient age, a key determinant of ovarian reserve and fertility outcomes, which may have confounded the findings. There was also a substantial heterogeneity in study designs and ovarian stimulation protocols, including variations in the use of random-start regimens, letrozole-based approaches, and gonadotropins, limiting comparability across studies. And, most included studies focused on surrogate markers such as oocyte yield, AMH levels, and antral follicle counts, without reporting long-term reproductive outcomes like pregnancies or live births, thereby restricting the clinical applicability of the results. Additional limitations of the study include reliance on retrospective studies from different geographic regions, which carry a risk of selection bias, and the absence of data on long-term reproductive outcomes (pregnancies, live births).

Future research must rectify these deficiencies by including more homogenous patient cohort and examining long-term reproductive outcomes especially pregnancy rates and live-birth rates. Studies are also needed to identify the molecular and clinical processes by which BRCA mutations and HR status affect fertility. Furthermore, there is a need for establishing consistent protocols for ovarian stimulation in BC patients with varying receptor and mutation profiles. Research should focus in developing the best ovarian stimulation protocol for optimal fertility outcomes in these patients.

## Conclusions

This systematic review and meta-analysis reveals that BRCA mutations seems to be associated with considerably diminished mature oocyte production during fertility preservation in BC patients. On the contrary, ER-positive status was associated with high AFC and oocyte yield indicating a more advantageous ovarian response. The present findings are from a limited number of heterogenous studies and hence must be interpreted with caution.

## Data Availability

Publicly available datasets were analyzed in this study. This data can be found here: PubMed, Embase, Scopus, and Web of Science databases.
